# Advancing Psychiatric Education: Leveraging Simulated Patients and Actors at the University of Pécs “Shame dies when stories are told in safe places”

**DOI:** 10.1192/j.eurpsy.2024.391

**Published:** 2024-08-27

**Authors:** J. D. Fekete, M. Simon, V. Voros, T. Tényi, A. Hambuch, A. Nagy

**Affiliations:** ^1^Department of Languages for Biomedical Purposes and Communication; ^2^Department of Psychiatry and Psychotherapy, University of Pécs, Medical School, Pécs, Hungary

## Abstract

**Introduction:**

The integration of simulated patients and actors (SPs) into psychiatric education has long been recognized as a transformative pedagogical approach, yielding substantial benefits to healthcare students and professionals.

**Objectives:**

The aim of this investigation was to evaluate the SP methodology and to refine it for future implementation in psychiatric education at the University of Pécs, Hungary.

**Methods:**

To investigate the feasibility and utility of incorporating SPs into psychiatric education, we conducted a preliminary study involving participants from the German Program in the University of Pécs, Hungary. This group consisted of 16 medical students in their 5th year of study. The study design involved participants forming groups of three, engaging in psychiatric interview with SPs. After the interview, SPs provided feedback from patient’s perspective, articulating their emotional responses. These sessions lasted 60 to 90 minutes.

**Results:**

Study participants expressed a range of apprehensive feelings, including inadequacy, the desire for correct performance, and acknowledgment of the emotional challenges involved. Another recurring issue was the students’ initial confidence contrasting with their later realization of subpar performance. A subset of students voiced concerns related to performance anxiety, particularly in light of being observed. Nevertheless, by the culmination of the course, students spontaneously recognized and valued the enriching nature of the experience. Pre-existing skills have been confirmed authentically by the feedback of the SP.

**Conclusions:**

Psychiatry, given its intricate and sensitive nature, necessitates a secure and controlled learning environment. SPs precisely provide this environment, facilitating the exploration of a broad spectrum of psychiatric disorders, emotional states, and patient interactions, all while upholding patient safety and confidentiality. Additionally, this methodology promotes the development of essential skills, including empathetic communication, the cultivation of therapeutic relationships. Moreover, the adaptability of SPs enables the creation of diverse scenarios reflecting real-world practice. Our preliminary findings and student feedback have provided a promising foundation for the design of a forthcoming pilot program in the next academic year. The integration of SPs into psychiatric education presents a dynamic, immersive, and highly effective approach, capable of markedly enhancing the quality of training.

**Disclosure of Interest:**

None Declared

